# Provider-Initiated Family Planning Within HIV Services in Malawi: Did Policy Make It Into Practice?

**DOI:** 10.9745/GHSP-D-19-00192

**Published:** 2019-12-23

**Authors:** Erin K. McGinn, Laili Irani

**Affiliations:** a The Palladium Group, Washington, DC, USA.; b Population Council, New York, NY, USA.

## Abstract

Malawi’s guidelines for *Clinical Management of HIV in Children and Adults* promote the practice of provider-initiated family planning (PIFP) for all clients over the age of 15. The guidelines recommend that providers should offer all clients condoms, offer injectables to female clients, and refer clients to another provider or site if clients prefer another family planning method. This study assessed to what extent family planning services had been integrated into HIV services among 41 facilities in Malawi (hospitals, health posts, health centers; public and nonprofit private) and how the reproductive rights of people living with HIV were being addressed. Data were collected through facility audits (N=41), provider interviews (N=122), client exit interviews (N=425), and mystery client visits (N=58). This study found that contrary to clinical protocols, only 14% of clients at the antiretroviral therapy (ART) clinic had reported being asked about their family planning/fertility intentions during the visit that day. Only 24% of providers at the facility had received training on family planning-HIV integration, and 21% had no family planning training at all. Overwhelmingly, ART clients relied on condoms to meet their family planning needs. Only 24% of ART clinics had injectables available, and only 15% of ART clinics had a full range of family planning methods (short- and long-acting, hormonal and non-hormonal) available to clients. These findings suggest that Malawi’s strong national policies on family planning-HIV integration, and specifically PIFP, are not being implemented in practice and thus not adequately addressing the family planning needs of clients with HIV. To improve PIFP, Malawi requires targeted systems changes. Facilities need to broaden their family planning method mix offerings. Furthermore, providers need more training on family planning and the importance of HIV clients having access to family planning services, and referral services need to be strengthened so providers can ensure clients have access to their method of choice in a timely manner.

## Abstract (precis)

Four years after Malawi embraced a policy of provider-initiated family planning (PIFP) within its HIV Clinical Guidelines, this policy remained largely unimplemented at the health facility level. Strengthening PIFP in Malawi’s public and private health facilities will require targeted and comprehensive systems changes.

## INTRODUCTION

### What Is Provider-Initiated Family Planning?

Provider-initiated family planning (PIFP) is an approach that encourages health care providers to routinely and proactively ask about a client’s reproductive intentions during the client-provider interaction, even if the client has come for other health services. The provider asks the client whether he/she intends to have another child (ever or in the near future) and what they might be using to space or avoid an unintended pregnancy. The provider then counsels the client on family planning and offers and/or refers the client for a range of contraceptive methods (or may provide information or referral for the client’s partner/spouse as needed).^1^ PIFP is rights-based, and the client can decline counseling and/or contraceptive methods as desired. The purpose of PIFP is to ensure there is “no missed opportunity to offer family planning.”^2^ Furthermore, a comprehensive review of over 2,500 articles shows that promoting voluntary family planning as part of routine HIV services is the number one evidence-based practice on how to meet the sexual and reproductive health needs (and rights) of women living with HIV.^3^

PIFP mimics the successful strategy of provider-initiated counseling and testing (PICT). In 2007, the World Health Organization established global guidelines on PICT for HIV, which encourages providers to routinely offer HIV testing as part of standard medical care for all patients in the context of a generalized HIV epidemic.^4^ PICT takes an “opt-out” approach, in that clients must specifically decline the test. Studies have shown a positive correlation between PICT and an uptake in HIV testing and condom use; therefore, as a programmatic intervention, PICT has been instrumental in increasing the number of people being tested for HIV and using condoms for HIV prevention.^5^

Numerous studies have shown that women and couples with HIV have a high unmet need for either limiting childbearing or delaying conception until their health or other personal circumstances improve.^6^^–^^8^ A recent 2016 study of pregnancy intentions of 220 women with HIV on antiretroviral therapy (ART) in a district hospital in Lilongwe, Malawi, found that 75% of them reported their current pregnancy was unintended (16% mistimed and 59% unwanted).^9^ HIV clients, like everyone else, deserve comprehensive family planning counseling and services and access to a full range of contraceptive methods to meet their reproductive intentions and life context, which likely change over time.

### Policies and Guidelines in Malawi

Malawi has a high prevalence of HIV. The 2015 Malawi Demographic and Health Survey (DHS) estimated an overall HIV prevalence of 8.8%, with a 10.8% prevalence among women and a 6.4% prevalence among men. Supplement 1 contains details on relevant indicators pulled from the last 2 rounds of the Malawi DHS and the findings from this study. Given this high prevalence and a growing number of patients seeking ART services, providing family planning services within ART clinics can be an effective way to address unmet need for contraception.

In 2011, the Government of Malawi issued its integrated guidelines for providing HIV care in its primary health care services, called *Clinical Management of HIV in Children and Adults*. These guidelines recommended PIFP be practiced at every scheduled visit at pre-ART follow-up and in ART clinics.^10^ Providers are instructed to assume all patients 15 years and older are sexually active and to proactively offer (30 or more) condoms to all men and women and injectables to women 15 years and older. The guidelines do take a rights-based approach in acknowledging that all patients with HIV have a right to a full reproductive life and should have the information on all family planning methods and be free to choose any family planning method of their choice. The guidelines further emphasize the need for providers to give clients an opportunity to refuse either method and to refer clients for counseling or other family planning methods (Box). Malawi updated these guidelines in 2014, with no substantial changes to the PIFP section.

BOX Malawi National Clinical Guidelines Instructions for Providers on Provider-Initiated Family Planning

- Assume all patients aged 15 years and older are sexually active.
- Offer condoms to all men and condoms and Depo-Provera [injectables] to all women.
- Give patients the opportunity to refuse either method.
- Refer clients to family planning clinics for further counseling or for other family planning methods.

Source: Section 6.5.1 of Malawi’s 2014 *Clinical Management of HIV in Children and Adults*.

The findings reported in this article are part of a larger multipronged assessment on the integration of family planning and HIV services in Malawi, undertaken in 2014 and 2015 by the United States Agency for International Development (USAID)-funded Health Policy Project.^11^ This study asked several implementation-related questions to ascertain whether Malawi’s clinical guidelines on PIFP were functioning at the facility level, including:

- What was the availability of family planning information and commodities within ART services?
- How are services organized to promote family planning-HIV integration?
  - Are the health workers providing HIV services trained in family planning?
  - For clients who need referrals, do providers know about referral points, i.e., other times or locations to which clients can be referred to obtain additional (family planning) services?
- Do clients on ART need and want family planning services?
- Do providers offer family planning to or initiate family planning with ART clients?

The objective of this article is to highlight the extent to which PIFP was being implemented per government guidelines, describe some system barriers to PIFP implementation, and provide some key recommendations to improve PIFP.

## METHODS

### Study Setting

Quantitative and qualitative data were collected between April and May 2015 from 41 facilities across 9 districts in Malawi—3 districts from each of the Northern, Central, and Southern Regions (Table 1). The districts were stratified by region and then randomly selected. A purposive sample of public and private facilities was chosen to represent a range of facility types. These included public health posts, centers, or clinics, which provided primary health care, and public hospitals, which offered in- and outpatient services at the local, district, and regional levels. Six additional public facilities were designated “integrated” health centers and were receiving targeted support from the United Nations Population Fund (UNFPA) to fully integrate primary health care. UNFPA funded the implementation of services in these facilities, including ensuring adequate stocks of drugs and providing in-service training. Seven private facilities were part of the Christian Health Association of Malawi (CHAM), which is a network of church-owned health facilities and hospitals, and the largest private provider of health care in Malawi. Two of the 7 CHAM facilities were Catholic and did not provide modern contraceptives.

**TABLE 1.** Types of Facilities From Which Data Were Collected Across 9 Districts in Malawi, by Facility Type, April–May 2015

Type of Facility	No.
Health post/center/clinic	19
Public hospital	9
Christian Health Association of Malawi hospital/health center	7
Integrated health center	6
Total number of facilities	41

The health centers/posts tended to be lower-volume sites staffed by 1 or 2 providers offering a range of primary health services (fully integrated), whereas the rural or urban hospitals tended to be higher-volume sites where specific services were provided by different health care workers. There was a great deal of variety of how family planning and HIV services were organized among all the facilities, and in many cases, facilities were using more than 1 model of family planning-HIV service integration. The government of Malawi had several policies, guidelines, and strategies that addressed integration of family planning into HIV services, but did not explicitly dictate models of integration;^12^ hence, a variety of models were being implemented.

Facilities in Malawi varied greatly in how they organized family planning and HIV services.

The approaches to service delivery integration included models where: (1) a client received multiple services from 1 provider in 1 room (often observed at UNFPA-supported integrated health centers), (2) the client might be seen by more than 1 provider in the same room or clinic space (often observed at health centers), (3) the client could access HIV and family planning services in different spaces (clinics) by different health care providers, but on the same day on the same facility grounds (often observed at public hospitals), or (4) completely non-integrated services where the client could only access HIV and family planning services on different days at that facility (often noted at health posts/clinics), or (5) needed to be referred elsewhere for their desired family planning services (in the case of CHAM facilities, or where clients wanted a family planning method not available at that site). Many facilities did not provide a range of family planning services/methods. Instead, Banja La Mtosogolo (a Marie Stopes International affiliate) would provide outreach services at these facilities at intermittent intervals (e.g., once per month) for clients interested in long-acting and permanent methods.

### Study Design

#### Data Collection

Both quantitative and qualitative data were collected between April and May 2015 using the following methods.

**Facility Audits.** At the 41 facilities, data collectors used a checklist to note the facility structure, observe patient flow in the ART clinic, observe the counseling and treatment spaces in the absence of clients, and note informational materials available at the site on the day of the data collection visit. The audits included a review of basic supplies/commodities at the ART clinic and a review of the ART registers where information on the clients who had been to the ART clinic that day were being registered (N=41).

**Staff Interviews.** Data collectors interviewed the facility in-charge (N=41) and up to 3 health care providers responsible for providing HIV services (N=122). The facility in-charges included doctors, registered nurses/midwives, clinical officers, and paramedical workers. The health care providers responsible for providing HIV services were nurses, midwives, clinical officers, health surveillance assistants, and paramedical workers, which included auxiliary nurses, medical assistants, or a nurse-midwife technician. On the day of the interview, the providers serving clients with HIV were first approached and interviewed. These providers cater to a range of HIV services including dealing with communicable diseases/tuberculosis, pediatric patients with HIV, and HIV counseling and testing (upon receiving referrals). A maximum of 3 providers per facility were interviewed.

**Client Exit Interviews.** Clients attending the ART clinic were invited to participate in the study after their regular visit for the day. Women ages 18–49 and men ages 18–59 who could read and write met the inclusion criteria. Literacy was an inclusion criteria to be able to sign the consent form. Even though the clients were randomly selected, we oversampled the number of women (compared to men) interviewed at each facility to better understand the needs and patterns of contraceptive use among patients with HIV. Based on the national prevalence of unmet need for family planning of 26% (DHS, 2010), we note that a sample size of 400 clients gives us statistical power with 95% confidence level and 5% margin of error to measure differences in family planning use among ART clients. Data collectors administered an exit interview to 425 clients.

**Mystery Clients.** To obtain a better understanding of client-provider interactions and referral mechanisms, 9 mystery clients (3 per region: 2 female and 1 male) were deployed to 20 facilities on days the data collection team was not visiting. These clients presented themselves as transfer patients with HIV seeking antiretrovirals (ARVs) and were trained to document whether they were spontaneously counseled and offered family planning (received PIFP as per the Government of Malawi clinical guidelines). If the health care provider did not offer family planning (i.e., if there was no provider initiation of family planning during ART services), the mystery clients were then to ask about family planning and document the provider’s response. Likewise, if the provider offered condoms and/or injectables, the mystery clients were also trained to ask about another family planning method and to document how the provider responded (e.g., did the provider offer a referral). Nine individuals made 58 mystery client visits to 20 facilities across all 3 regions. The mystery clients presented themselves as ART clients temporarily in the area and in need of ARV resupply (e.g., visiting a sick relative, husband just transferred). The female mystery clients were 20–36 years old. The male mystery clients were 19, 33, and 35 years old.

The mystery clients were trained to first see whether providers mentioned family planning (PIFP), and if not, to ask about it. They were provided with suggestions for different profiles or scenarios regarding their reproductive intentions. For example, the older women said they had 3 or 4 children and didn’t want any more, whereas younger women were told to say they had 1 child and wanted to space their births. The 19-year-old man presented himself as a student. The research team (with knowledge of the Ministry of Health) created temporary health passports for the clients to support their profile. Any ARVs collected by the clients were documented and returned to the health system via the Lighthouse Clinic in Lilongwe.

#### Data Entry, Cleaning, and Analysis

Quantitative data from facilities were collected using paper data collection forms, then entered into templates developed in CSPro6.1 and exported into STATA10 for analysis. Qualitative data were transcribed and then translated into English.

#### Ethical Considerations

The study received ethical approval from Malawi’s National Health Sciences Research Committee in Lilongwe, Malawi, and the Institutional Review Board of Health Media Lab in Washington, DC, USA. Interviews were conducted in a private space and lasted under 1 hour. All participants (facility in-charges, providers, and clients) were provided details of the study in advance, and read aloud the consent form, which they then signed. No names were recorded, only titles, or in the case of clients, basic sociodemographic data. Providers and clients at the facilities were not given any compensation for their participation in this study. All informed consent information and subsequent questionnaires were translated and administered in one of the prevalent local languages of the region: Chichewa, Chitumbuka, or Yao.

## RESULTS

### Availability of Family Planning Information and Commodities

**FIGURE 1.**
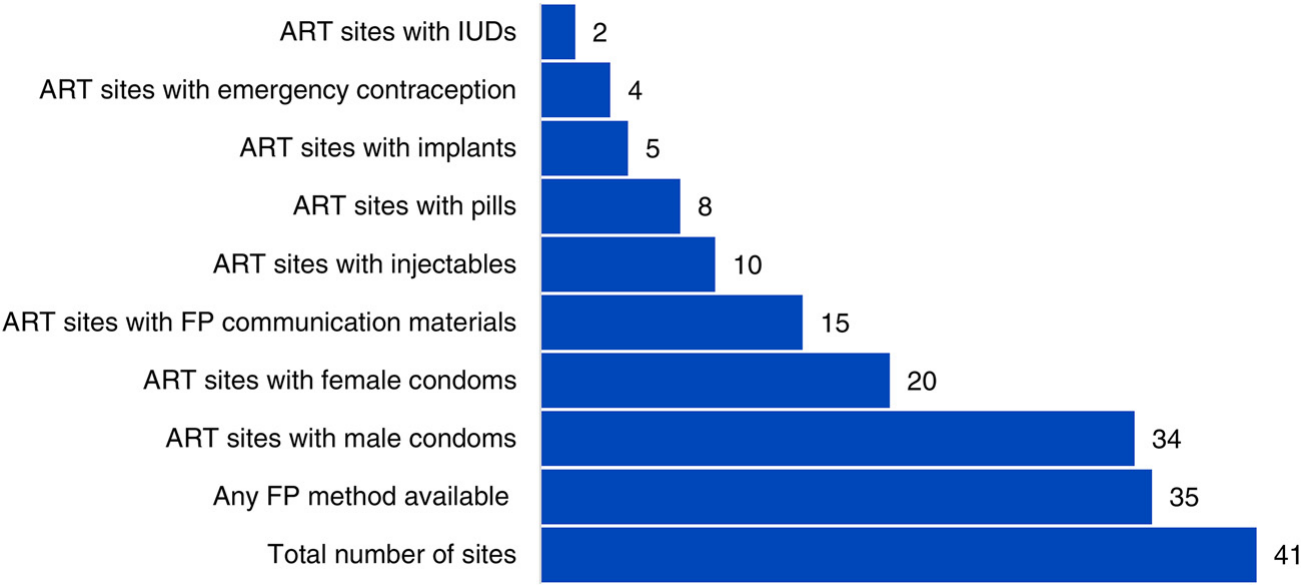
Availability of Family Planning Methods in 41 Facilities in 9 Districts in Malawi, April–May 2015 Abbreviations: ART, antiretroviral therapy; FP, family planning; IUD, intrauterine device.

Of the 41 facilities with an ART clinic or an outpatient department where ART services were being provided, 85% (n=35) had family planning methods available, but this was mainly because most facilities had condoms available, which counts as a family planning method even if primarily used for prevention of sexually transmitted infections, including HIV. Very few facilities had a variety of family planning methods available. Of the 35 facilities where family planning was available, 10 of those sites (24%) had injectables and condoms. Only 5 sites offered implants and only 2 offered intrauterine devices (IUDs). Six of the facilities (15%) had a range of hormonal, non-hormonal, and short- and long-acting contraceptives available. Furthermore, only 15 (37%) of ART clinics had family planning information materials displayed (Figure 1).

Of the 35 facilities where family planning was available, 6 had a range of hormonal, non-hormonal, and short- and long-acting contraceptives available.

### Organization of Services

**FIGURE 2.**
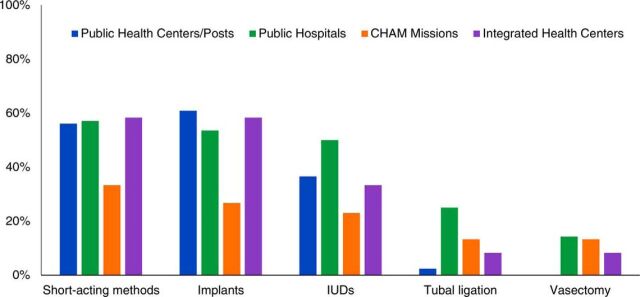
Percentage of Providers in 9 Districts in Malawi Who Reported Receiving Family Planning Training on Specific Contraceptive Methods, by Method and Facility Type, April–May 2015 (N=96) Abbreviations: CHAM, Christian Health Association of Malawi; IUD, intrauterine device.

Interviews with 122 health care providers found that 93% had received training on HIV services, but only 79% had received any training in family planning, and only 24% of the health providers had received any training specifically on integration of family planning and HIV services. In addition, fewer providers working in CHAM facilities had received family planning training (only 71% had been trained in family planning compared to 76%–88% of the providers in other facilities), and of those who received family planning training, fewer had received training in short-acting methods, implants, or IUDs compared to the providers at the other facilities. Overall, 53% of the providers with some pre- or in-service family planning training had been trained on short-acting methods, 38% on IUDs, and 21% of those in cadres eligible to provide sterilization had been trained on permanent family planning methods (Figure 2).

When health care providers were asked if the ART services had received some reorganization to accommodate family planning services, 83% of providers said yes (Table 2). When asked about different ways services had been reorganized, 42% of providers said that ART protocols had been revised. On-site protocols are copies of operational guidelines for each type of facility within which the HIV clinical guidelines are incorporated. As well, 51% of providers said that they had established informal referral agreements between their ART clinic and those providing family planning services either within the facility or at a public facility nearby providing family planning services. Only 15% of providers mentioned that ART provision time was adjusted to accommodate family planning, and 11% of providers reported that the ART registers had been revised.

**TABLE 2.** Description of Organization of Antiretroviral Therapy and Family Planning Services, According to the Health Service Provider Within Selected Facilities, by Facility Type, Malawi

	Health Centers/Posts No. (%)	Public Hospitals No. (%)	CHAM Missions No. (%)	Integrated Health Centers No. (%)	Total No. (%)
**Total number of providers**	54 (44.3%)	32 (26.2%)	21 (17.2%)	15 (12.3%)	122 (100%)
**Have ART services been reorganized to accommodate FP services?**					
Yes	43 (79.6%)	26 (81.3%)	17 (80.9%)	15 (100%)	101 (82.8%)
No	11 (20.4%)	6 (18.8%)	4 (19.0%)	0	21 (17.2%)
**How have ART services been reorganized to accommodate provision of FP services?^a^**					
Created more space	8 (18.6%)	4 (15.4%)	3 (17.7%)	4 (26.7%)	19 (18.8%)
Revised ART on-site protocols to accommodate FP services	12 (27.9%)	17 (65.4%)	5 (29.4%)	8 (53.3%)	42 (41.6%)
Trained ART providers in different FP methods	21 (48.8%)	11 (42.3%)	4 (23.5%)	12 (80.0%)	48 (47.5%)
Created informal referral agreements within facility	26 (60.5%)	14 (53.8%)	6 (35.3%)	5 (33.3%)	51 (50.5%)
Developed facility referral agreements across facilities	14 (32.6%)	10 (38.5%)	5 (29.4%)	2 (13.3%)	31 (30.7%)
Revised ART client registers to accommodate FP services	4 (9.3%)	4 (15.4%)	1 (5.9%)	2 (13.3%)	11 (10.9%)
Adjusted operating time for ART services	7 (16.3%)	2 (7.7%)	2 (11.8%)	4 (26.7%)	15 (14.9%)
Provided ART/FP on same day	3 (6.9%)	3 (11.5%)	1 (5.9%)	0	7 (6.9%)
**Do you have time/opportunity to counsel ART clients on FP methods?**					
Yes	51 (94.4%)	29 (90.6%)	20 (95.5%)	14 (93.3%)	144 (93.4%)
No	2 (3.7%)	2 (6.3%)	1 (4.8%)	1 (6.7%)	6 (4.9%)
Not sure	1 (1.9%)	1 (3.1%)	0	0	2 (1.6%)
**What FP methods do you counsel ART clients on?^a^^,^^b^**					
Fertility awareness methods	51 (100%)	29 (100%)	20 (100%)	14 (100%)	144 (100%)
Pills	44 (86.3%)	21 (72.4%)	18 (90.0%)	12 (85.7%)	95 (83.3%)
Male condoms	51 (100%)	26 (89.7%)	20 (100%)	14 (100%)	111 (97.4%)
Female condoms	45 (88.2%)	25 (86.2%)	19 (95.0%)	13 (92.9%)	102 (89.5%)
Injectables	47 (92.2%)	26 (89.7%)	20 (100.0%)	14 (100%)	107 (93.9%)
IUD	28 (54.9%)	18 (62.1%)	12 (60.0%)	5 (35.7%)	63 (55.3%)
Implants	43 (84.3%)	19 (65.5%)	16 (80.0%)	10 (71.4%)	88 (77.2%)
Female sterilization	34 (66.7%)	20 (69.0%)	11 (55.0%)	7 (50.0%)	72 (63.2%)
Male sterilization	25 (49.0%)	13 (44.8%)	7 (35.0%)	5 (35.7%)	50 (43.9%)
Emergency contraception	19 (37.3%)	17 (58.6%)	10 (50.0%)	5 (35.7%)	51 (44.7%)
**Are clients referred out for services?**					
Yes	48 (88.9%)	15 (46.9%)	16 (76.2%)	12 (80.0%)	91 (74.6%)
No	6 (11.1%)	17 (53.1%)	5 (23.8%)	3 (20.0%)	31 (25.4%)
**What prior knowledge do you have of facilities to which you are referring clients for FP services?^a^^,^^c^**					
Services provided	32 (66.7%)	10 (66.7%)	12 (75.0%)	9 (75.0%)	63 (69.2%)
Weekdays on which services are provided	34 (70.8%)	11 (73.3%)	6 (37.5%)	7 (58.3%)	58 (63.7%)
Times when services are provided	25 (52.1%)	5 (33.3%)	5 (31.3%)	5 (41.7%)	40 (44.0%)
Transport costs to reach the referral site	14 (29.2%)	3 (20.0%)	5 (31.3%)	4 (33.3%)	26 (28.6%)
No prior knowledge	5 (10.4%)	5 (33.3%)	1 (6.3%)	2 (16.7%)	13 (14.3%)

a Categories are not mutually exclusive.

b Of the 114 providers who counsel ART clients on FP.

c Of the 91 providers who refer clients for services.

Abbreviations: ART, antiretroviral therapy; CHAM, Christian Health Association of Malawi; FP, family planning.

At the time of data collection, ART registers were supposed to already have 3 columns to indicate whether family planning counseling, condoms, and/or injectables were provided. These columns were added after the clinical guidelines were first introduced. Further, this was mandated by the HIV & AIDS Department of the Ministry of Health as injectables and condoms were being dispensed through the ART essential drug list. This study investigated whether there were any other columns added to the ART registers corresponding to additional family planning methods to determine whether any additional methods beyond condoms or injectables were provided to clients on ART. When data collectors requested to view the registers at ART clinics, half (17) were unavailable—either providers would not allow data collectors to review, the register was not yet out of the locked cabinet for the day (despite patients being seen), there was a shortage of registers at the clinic, or it was at another location (or lost/misplaced). Of the ART registers reviewed at 18 sites, 6 had extra columns added in the ART register to document family planning provision, whereas another 8 maintained a separate family planning register. The remaining 4 facilities had no mechanism to document additional family planning service provision (beyond condoms or injectables) at the ART clinic. There are no other documents that capture information about clients coming to the facility to receive ART medication. Malawi’s Department of HIV & AIDS regularly monitors the number of condoms and injectables distributed to ART clients.

Nonetheless, 93% of providers reported they had the time and opportunity to counsel ART clients on family planning methods available to them (Table 2), and almost all mentioned condoms and injectables as the methods they counseled on. Supplement 2 details precise wording for questions. Fewer providers mentioned counseling on implants (77%), IUDs (55%), female sterilization (63%), or vasectomy (44%) (Table 2). About 75% of providers mentioned they referred clients for family planning to other facilities, but most lacked knowledge of details about when those services were available or the transport costs to those services (Table 2).

### Clients’ Reproductive Intentions and Contraceptive Needs

We conducted 425 exit interviews with HIV clients (at least 10 per site across all 41 sites). Of these clients, 419 of them disclosed they were HIV-positive: 332 were female, and 87 were male. Of the female clients interviewed (n=332), 17 (5%) were currently pregnant, and most reported the pregnancy as either mistimed (n=9) or unwanted (n=4). Of the 315 female clients who were not pregnant, 52% reported not wanting any more children, 14% wanted to wait more than 2 years to get pregnant, and an additional 25% were unsure. Fifty clients (16%) reported already using sterilization as their family planning method.

Of the total number of male and female clients eligible to use family planning (i.e., themselves or partner not pregnant, not already sterilized, n=358), 60% were using a method to avoid pregnancy (this is comparable to the modern contraceptive prevalence of married women in Malawi reported in the 2015/16 DHS of 58%). Half were using male condoms, and one-third were using injectables. Only 11% were using implants, about 4% were using female condoms, 4% were using pills, and only 1 client (0.5%) was using an IUD.

When asked about their preferences for receiving integrated services, 97% of clients interviewed preferred receiving fully integrated services (i.e., in the same clinic/same room, same day). In addition, 90% of clients said that they would be willing to wait longer to get multiple services per visit. Over three-quarters of clients stated making fewer trips to the facility as the benefit of receiving integrated family planning-HIV services, and 43% cited reduced travel costs as a benefit of integration. Less than 10% of clients mentioned reduced stigma as a benefit of integrating services, even though theoretically, integrated services can reduce HIV-related stigma because other clients and providers would not explicitly know the nature of the service the client was seeking.

Clients cited fewer trips to facility, reduced travel costs, and reduced stigma as benefits of integrated family planning and HIV services.

Only 14% of clients who accessed HIV services said that providers had asked them about fertility and offered family planning.

### Provider-Initiated Family Planning

To ascertain whether PIFP was being implemented in ART clinics as stipulated in the national clinical guidelines, we specifically asked clients who had come for ART (n=355) and other HIV services (n=51) if any health provider had asked them about their fertility intentions and/or offered them family planning during their visit that day (Supplement 2, Question 2). Only 56 (14%) said yes.

In another line of questioning, we asked clients if a provider had *ever* inquired about their fertility intentions (Supplement 2, Question 3). Of the 77 clients that said yes, 13 reported “every time,” 21 said “often,” 11 said “sometimes,” and 23 said “rarely.”

### Mystery Client Visits

Of 58 visits, only 2 of the mystery client visits reported the provider proactively offered family planning to the clients:

*She [the provider] noted my book had nothing on family planning and started advising me of family planning and all methods like vasectomy, Norplant [outdated term for implants], IUCD [intrauterine contraceptive device] … she later advised me to opt for a family planning method to avoid unwanted pregnancy.* — Female, 20, health center

*She [the provider] said “All of [the choices] are present,” and added it was my choice to choose which one I prefer.* — Female, 36, district hospital

Eleven mystery client visits at 7 different facilities resulted in the mystery clients being told that they could not receive these ART services since they were not registered as regular clients at the facility. For the most part, the mystery clients did not encounter a conducive or welcoming environment for PIFP. The young male mystery client reported not being taken seriously at 2 facilities when he asked about family planning and was only offered condoms:

*I then asked for family planning to which he [the provider] responded how come I wanted family planning when I was in school [provider offered family planning options and information, but laughed at him].* — Male, 19, health center

Another health center fared particularly poorly in their interaction with the mystery clients. The 2 quotes below are from the same location:

*When I asked him [the provider] about family planning he shouted at me saying the room was not for family planning: “Had it been that you are looking for family planning you could have gone to the family planning room. Go out, I want to assist other patients please.” I ask him about condoms. He said I am wasting his time, there was no condoms.* — Male, 33, health center

*Then I asked about family planning and I was told that I should not delay him [the provider] as he has a lot of work to do and he sent me away. He said that if I want family planning methods I should come the following day around 8 a.m.* — Female, 24, health center

## DISCUSSION

This study used a mixed-methods approach to triangulate and validate information from providers, clients, and facility audits in an effort to reveal whether PIFP in ART clinics was truly being implemented as envisioned by the 2011 (and 2014) clinical management guidelines in Malawi. For the most part, this service delivery approach has largely been unrealized in practice.

Although 93% of providers reported having enough time for counseling ART clients on family planning, only 14% of clients reported being asked about their fertility intentions or being counseled on family planning at the ART clinic that day. Using mystery clients is a valuable approach to obtaining information on client-provider interactions.^13^^,^^14^ It allows researchers to test how services are provided given certain client profiles, minimizes recall or other biases in self-reporting through interviews, and reduces the “Hawthorne Effect”—that data collectors undertaking observational assessments may influence provider and client interactions merely by their presence. Therefore, this study also conducted mystery client visits in a subset of the facilities. Only 2 of 58 (3%) mystery client visits resulted in PIFP being offered to the client.

Although 93% of providers reported having time to counsel clients on family planning, only 14% of clients reported being asked about their fertility intentions or being counseled about family planning.

The client exit interviews revealed a significant need for family planning services among clients with HIV. Our findings that the vast majority of female clients wanted no more children or wanted to delay childbearing for more than 2 years echo multiple other studies on the reproductive intentions of women with HIV.^6^^–^^8^ In particular, we found that 13 of 17 of the pregnant women reported the pregnancy as mistimed or unwanted. Although this was a small sample size, it echoes a recent article from Malawi on pregnant women on ART (N=220) that found 75% of women reported the pregnancy as mistimed or unwanted.^9^ Through client exit interviews, clients with HIV expressed a significant interest in receiving integrated services. Almost all said they would be willing to wait longer to receive multiple services to reduce trips to the facility and transportation costs. This finding suggests the financial and opportunity costs of seeking health care may be more onerous for Malawians than managers of the health system realize.

The demand for integrated family planning-HIV services and the high unmet need for family planning among women with HIV in Malawi underscores the need for more focused efforts to implement PIFP in Malawi’s HIV services. This will require more concrete programmatic interventions to strengthen and sustain family planning-HIV integration, as well as further investigation into the challenges providers and facility managers face in institutionalizing PIFP, such as time constraints and lack of training, inadequate organization of services, stock-outs, and insufficient accountability for implementing national guidelines. Exploring provider perspectives on family planning-HIV integration and possible biases or negative attitudes about PIFP would also be helpful to understand what additional interventions or trainings are needed. Although 93% of providers said they had enough time to discuss their clients’ family planning needs (Supplement 2, Question 1) our triangulation of data from exit interviews, and mystery clients suggest that clients only see providers for less than 5 minutes on average. Thus, the authors conclude that the providers’ response of adequate time was likely due to a social desirability bias. It is also possible that providers at ART clinics are not motivated to provide PIFP. Facilities/providers currently implementing PIFP focus largely on male and female condoms and injectables, which certainly is the emphasis of the national clinical guidelines. Furthermore, it is not surprising that providers focus on condoms and injectables because providers are likely conditioned to promote condoms (for HIV prevention), and in addition to being part of the essential ART drug list, injectables are the most common family planning method in Malawi. Providers need to be able to counsel clients with HIV on a full range of family planning methods, provide some method choice, and effectively refer as needed. This study found that although 77% of the providers reported counseling on pills or implants, fewer mentioned female sterilization (63%), IUDs (55%), or vasectomy (44%). This study also found only about a third of facilities had injectables available where ART services were provided, and family planning communications materials were also scarce. This lack of method availability affects informed choice. However, HIV clients, like everyone else, deserve comprehensive family planning counseling and services and access to a full range of methods to meet their reproductive intentions and life context, which likely change over time.

The demand for integrated family planning-HIV services and the high unmet need for family planning among women with HIV underscores the need for more focused efforts to implement provider-initiated family planning in Malawi’s HIV services.

### Limitations

We note the following limitations of this study. Response bias may have affected our data collected from in-person interviews. Facility in-charges and providers may have overstated the level of family planning-HIV integration of services and availability of time for counseling or under-estimated stock-outs. Clients in exit interviews may have been subject to recall bias. Furthermore, the presence of data collectors at the facility conducting interviews and conducting the facility audit may also have produced a Hawthorne effect with providers and clients, affecting service delivery approaches, though no direct observation of client-provider interactions occurred. Our use of mystery clients encountered some challenges we did not anticipate. The mystery clients faced challenges in being served at facilities due to presenting as ad hoc or “emergency” clients, and a few were refused services outright.

## CONCLUSION

Although Malawi should be recognized as an early adopter of PIFP within its HIV-management guidelines, 4 years after adopting these guidelines, implementation of PIFP was largely unrealized at the clinic level. The results of the larger study on which this article is based were shared with the Ministry of Health and USAID in-country at a national conference and to a wider audience of implementers and researchers in 2 global meetings and conferences. Informal follow-up with in-country contacts during the drafting of this article suggests that since the collection of data reported in this study, the Government of Malawi has conflicting information on integrating family planning into HIV at the policy level. For example, in 2016, Malawi issued a third edition of its *Clinical Management of HIV in Children and Adults,* which maintained PIFP as a protocol during ART services. However, the 2016 update to the ART registers removed columns to report condom or injectable provision. As such, it is likely that this article’s findings on PIFP at the facility level remain applicable and further institutionalizing of PIFP in Malawi’s public and private health facilities requires targeted and comprehensive systems changes. Only about a quarter of providers said that they had been trained on family planning-HIV integration, and only half had information on where to refer clients for their family planning needs. These findings suggest that providers may not be fully aware of how to implement PIFP; hence further training may be warranted. Simple job aids to reinforce PIFP and support referrals may also be helpful. Routine follow-up of patients during subsequent visits by their HIV service providers to see if they are having their family planning needs met, and a more robust provision of a full range of family planning methods accessible to each client either within the ART clinic or through a referral, would also help support HIV clients to meet their reproductive intentions. In addition, facility in-charges and health management teams should be held accountable to measurable PIFP indicators. This can be done through the routine review of data among hospital staff and open dialogue on how to make family planning services more available to ART clients. Specifically, there is a need for improved tracking and reporting of family planning commodities and services provided through ART services, expanded family planning method choice where possible, and strengthened referral systems. Continuous availability of family planning commodities at ART clinics coupled with a formalized referral system will allow the ART services to be more integrated and hence meet the needs of ART clients. Finally, community sensitization and demand-creation for family planning-HIV integrated services might help inform clients with HIV about their reproductive health rights and empower them to proactively ask for family planning during their visits to the clinic; this may also help to promote more accountable health facilities where PIFP is not being practiced.

## Supplementary Material

19-00192-McGinn-Supplement1.docx

19-00192-McGinn-Supplement2.docx

## Notes

**Peer Reviewed**

**First Published Online:** November 27, 2019
