# Supplementary material for: Provider-Initiated Family Planning Within HIV Services in Malawi: Did Policy Make It Into Practice?
Source: Glob Health Sci Pract. 2019 Dec 23;7(4):540–50. doi: 10.9745/GHSP-D-19-00192 (PMC6927829; doi:10.9745/GHSP-D-19-00192)
Supplement: 19-00192-McGinn-Supplement1.docx [file 19-00192-McGinn-Supplement1.docx]

**Supplement 1: Key Indicators from the 2010 and 2015/16 Malawi Demographic and Health Surveys**

|  | DHS 2010 | DHS 2015/16 | Reported in current study |
| --- | --- | --- | --- |
| National TFR (number of children per woman) | 5.7 | 4.4 | N/A |
| Currently married women using modern contraception (mCPR) | 42.2% | 58.1% | 56.4%^a^ |
| Unmet Need (married) | 26.2% | 18.7% | N/A |
| Currently pregnant | 9.0% | 7.6% | 5.1% |
| Pregnancies in past 5 years mistimed or unwanted | 44.3% | 40.9% | N/A |
| Current pregnancy mistimed or unwanted |  |  | 76.5% |
| HIV prevalence (all) | 10.6% | 8.8% | N/A |
| HIV prevalence (women) | 12.9% | 10.8% | N/A |
| Wants no more children (women)^b^ | 46.9% | 49.2% | 52% |
| Using sterilization (male and female) | 9.8% | 11% | 16% |
| Wants another child later (women) | 36.3% | 32.9% | 14% |
| Using LARCs | 1.6% | 12.6% | 11.5% |
| Unsure about timing of next pregnancy or if they want another one | 3.0% | 6.1% | 25% |

^a^ All female clients, regardless of marital status.

^b^ DHS indicator “Percentage of currently married or in union women who want no more children or are sterilized.”

Abbreviations: LARC, long-acting reversible contraceptive; mCPR, married contraceptive prevalence rate; TFR, total fertility rate.
