# Supplementary material for: Provider-Initiated Family Planning Within HIV Services in Malawi: Did Policy Make It Into Practice?
Source: Glob Health Sci Pract. 2019 Dec 23;7(4):540–50. doi: 10.9745/GHSP-D-19-00192 (PMC6927829; doi:10.9745/GHSP-D-19-00192)
Supplement: 19-00192-McGinn-Supplement2.docx [file 19-00192-McGinn-Supplement2.docx]

**Supplement 2: Select Interview Questions Posed to Providers and Clients in 9 Districts in Malawi, April-May 2015**

Question 1: The questions posed to providers were: “Do you have the time and opportunity to counsel ART clients on the various family planning methods available to them?” and “If Yes, what FP methods do you counsel them on?”

Question 2: The specific question asked in the exit interview was “If you came to the clinic for ART and other HIV services, did anyone ask you if you wanted to have more children and offer you family planning?”

Question 3: The survey question posed to the client was “At the ART clinic, has a provider ever inquired about your fertility intentions and counseled you on family planning?”
